# Fundamentals of protein interaction network mapping

**DOI:** 10.15252/msb.20156351

**Published:** 2015-12-17

**Authors:** Jamie Snider, Max Kotlyar, Punit Saraon, Zhong Yao, Igor Jurisica, Igor Stagljar

**Affiliations:** ^1^ Donnelly CentreDepartment of BiochemistryDepartment of Molecular GeneticsUniversity of TorontoTorontoONCanada; ^2^ Princess Margaret Cancer CenterIBM Life Sciences Discovery CentreUniversity Health NetworkOntarioCanada

**Keywords:** bioinformatics, interactome mapping, PPI technologies, protein‐protein interactions (PPIs), proteomics, Genome-Scale & Integrative Biology, Post-translational Modifications, Proteolysis & Proteomics

## Abstract

Studying protein interaction networks of all proteins in an organism (“interactomes”) remains one of the major challenges in modern biomedicine. Such information is crucial to understanding cellular pathways and developing effective therapies for the treatment of human diseases. Over the past two decades, diverse biochemical, genetic, and cell biological methods have been developed to map interactomes. In this review, we highlight basic principles of interactome mapping. Specifically, we discuss the strengths and weaknesses of individual assays, how to select a method appropriate for the problem being studied, and provide general guidelines for carrying out the necessary follow‐up analyses. In addition, we discuss computational methods to predict, map, and visualize interactomes, and provide a summary of some of the most important interactome resources. We hope that this review serves as both a useful overview of the field and a guide to help more scientists actively employ these powerful approaches in their research.

## The importance of studying PPIs

As the basic unit of life, cells represent complex biological entities, whose normal function revolves around a delicate interplay between multiple diverse biomolecular systems. Proteins are vital components of these systems, acting as molecular machines, sensors, transporters, and structural elements (among others), with interactions between proteins, hereinafter called protein–protein interactions (PPIs), being key to their function.

Protein–protein interactions are inherently dynamic in nature, adjusting in response to different stimuli and environmental conditions. This provides considerable flexibility in function and allows cells to adapt in a measured way to changing circumstances. Even a subtle dysfunction of PPIs can have major systemic consequences, perturbing interconnected cellular networks and producing disease phenotypes (Barabási *et al*, 2011). Developing in‐depth, dynamic PPI maps is therefore critically important in helping us comprehend these complex processes, and identify new proteins and PPIs suitable for therapeutic intervention.

Over the years, we have seen an emergence and growth of a wide range of exciting technologies for the identification and characterization of PPIs. Selecting “the best” technology for a given research application is thus non‐trivial. Here, we highlight the strengths and weaknesses of various methodologies, to aid in selecting the appropriate method for the problem at hand. Note that this review does not aim to cover all PPI methods; instead, we focus on newer approaches and earlier methods that remain widely used, and strongly impacted research.

## Key considerations

While numerous methods are available for the large‐scale study of PPIs, there is no one “perfect” method for all situations, and each has its own strengths and weaknesses. When selecting a suitable method to study interacting partners of a protein of interest, the following factors should be considered:

1. *The Goal of the Study* must be clearly defined. Discovery‐driven studies usually aim to explore interactomes in an unbiased manner on a proteome‐wide scale. In contrast, targeted interactome studies focus on a subset of PPIs and therefore confine themselves to smaller libraries or arrays corresponding to a defined set of candidate interaction partners. Different methods are better suited to certain classes of proteins as well as to formats and scales, and selection of one that best matches the research goals is critical.
2. *The Distinct Nature of the PPIs Being Studied*. All PPIs have intrinsic biophysical properties, giving each its own unique features. Some important characteristics to consider are the PPI “strength” (binding affinity), and whether the interaction is transient or stable (Perkins *et al*, 2010). Different bioassays display variable sensitivity, and although generally all can detect stable PPIs, only a fraction are capable of detecting transient interactions. It is also important to determine whether or not posttranslational modifications, co‐factors, or additional binding partners are required (e.g., a PPI may be mediated indirectly through a protein complex), as well as where in the cell interactions are expected to occur, since the selected assay must be compatible with these elements.
3. *Time/Cost Constraints*. Not all methods scale‐up equally, and some, while offering powerful advantages on a smaller‐scale, can become significantly more expensive and time‐consuming as the number of interactions studied increases. Additionally, the time and cost required to develop the necessary reagents (e.g., specific constructs, libraries) needs to be considered.
4. *Specialized Equipment and Expertise*. Finally, it is important to ensure that all necessary resources and knowledge required to fully take advantage of a particular method are available. Although the majority of methods are straightforward, some do require specific instrumentation and expertise. Most methods, especially those that attempt to study interactomes on a genomewide scale, also require strong bioinformatics support for analysis and data cleaning.

## Guide to available methods

While many PPI assays exist, we present below some of the newer and more widely used approaches, providing a concise overview of their key principles, advantages, and limitations. Key references for each technique, including examples of their large‐scale application, can also be found in Table 1.

**Table 1:** Useful literature references for protein–protein interactions (PPI) methods

Assay	Relevant literature reviewing or introducing technique	Examples of interaction studies using technique
Y2H	Hamdi and Colas (2012); Ferro and Trabalzini (2013); Stasi *et al* (2015)	Yu *et al* (2008); Weimann *et al* (2013); Rajagopala *et al* (2014); Rolland *et al* (2014); Grossmann *et al* (2015)
MYTH	Snider *et al* (2010); Petschnigg *et al* (2012)	Snider *et al* (2013); Lam *et al* (2015); Gulati *et al* (2015)
LUMIER	Blasche and Koegl (2013)	Barrios‐Rodiles *et al* (2005); Xu *et al* (2014); Taipale *et al* (2014); Sahni *et al* (2015)
MAPPIT	Sahni *et al* (2015); Lievens *et al* (2011); Lemmens *et al* (2015)	Lievens *et al* (2009); Bovijn *et al* (2013); Rolland *et al* (2014)
KISS	Lievens *et al* (2014)	Amano *et al* (2015)
BIFC	Kerppola (2008); Zhang *et al* (2015)	Lee *et al* (2011b); Snider *et al* (2013); Cooper *et al* (2015)
MaMTH	Petschnigg *et al* (2014)	**–**
BRET/FRET	Ciruela (2008); Xie *et al* (2011); Ma *et al* (2014)	Kocan *et al* (2008); Audet *et al* (2010); Mandić *et al* (2014); Sauvageau *et al* (2014)
AP‐MS	Dunham *et al* (2012)	Wang and Huang (2008); Babu *et al* (2012); Havugimana *et al* (2012)
BioID‐MS	Roux *et al* (2012)	Kim *et al* (2014); Dingar *et al* (2015); Lambert *et al* (2015)
PLA	Koos *et al* (2014)	Chen *et al* (2014)
LRC‐TriCEPS	Frei *et al* (2013)	Frei *et al* (2012)
AVEXIS	Sanderson (2008); Kerr and Wright (2012); Sun *et al* (2012)	Bushell *et al* (2008); Martin *et al* (2010); Crosnier *et al* (2011)

### The yeast two hybrid (Y2H)

#### Principle

**Figure 1:**
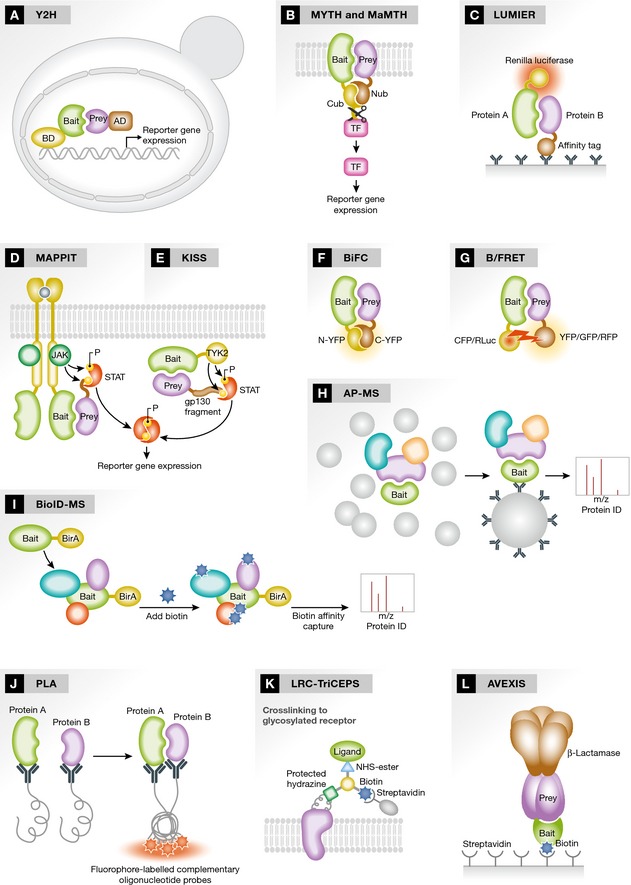
Overview of interaction proteomics technologies Schematic representations of selected newer and widely used PPI assays. (A) Yeast Two Hybrid (Y2H). (B) Membrane Yeast Two Hybrid (MYTH) and Mammalian Membrane Two Hybrid (MaMTH). (C) Luminescence‐based Mammalian Interactome Mapping (LUMIER). (D) Mammalian Protein‐Protein Interaction Trap (MAPPIT). (E) Kinase Substrate Sensor (KISS). (F) Bimolecular Fluorescence Complementation (BiFC). (G) Bioluminescence/Fluorescence Resonance Energy Transfer (B/FRET). (H) Affinity Purification‐Mass Spectrometry (AP‐MS). (I) Proximity‐dependent Biotin Identification Coupled to Mass Spectrometry (BioID‐MS). (J) Proximity Ligation Assay (PLA). (K) Ligand‐Receptor Capture‐Trifunctional Chemoproteomics Reagents (LRC‐TRiCEPS). (L) Avidity‐based Extracellular Interaction Screen (AVEXIS).

Originally developed 25 years ago (Fields & Song, 1989), the Y2H assay (Fig 1A) remains one of the most popular PPI methods. Y2H‐based systems can be used to detect interactions between two proteins, protein and nucleic acid, and also in small‐molecule screens (Hamdi & Colas, 2012; Ferro & Trabalzini, 2013). The classic Y2H involves the physical separation of two functional moieties of a transcription factor, specifically a DNA‐binding domain (BD) and a transcriptional activation domain (AD), and their fusion to candidate interacting proteins. If a protein bearing an AD interacts with, or comes in close proximity to, a protein bearing a BD, the AD and BD are able to function together as a transcription factor, and direct expression of a reporter gene (Fields & Song, 1989).

#### Advantages

The Y2H approach is simple, well established, and low cost and can be easily set up in most laboratory environments. Y2H is scalable and effective for use in both large‐scale screening studies, and smaller efforts investigating specific PPIs. Another benefit is that the assay is carried out *in vivo* in the context of the yeast cell, helping avoid some of the complications and artifacts associated with cell lysis. This assay is best suited for the detection of binary interactions (Hamdi & Colas, 2012; Ferro & Trabalzini, 2013).

#### Limitations

The use of a yeast host means that the PPIs from other organisms may in some cases not be detectable, due to poor expression, or a lack of necessary posttranslational modifications, cofactors, or other binding partners. The method requires that both interacting proteins access the nucleus (in order to drive transcription of reporter), which means that proteins confined to particular cellular environments (e.g., the membrane) cannot be studied in their full‐length form. The proteins used in this method are also often overexpressed, which can lead to non‐specific interactions. Altogether, these effects can lead to a high false‐positive rate, necessitating careful follow‐up analysis to identify true, biologically relevant interactions. The readout of this method is also indirect, preventing spatial or temporal analysis of PPIs (Hamdi & Colas, 2012; Ferro & Trabalzini, 2013).

### Membrane yeast two hybrid (MYTH)

#### Principle

The MYTH assay (Fig 1B) is designed for the analysis of the interactions of membrane proteins. It is based on a split‐ubiquitin approach, whereby the ubiquitin protein is divided into two distinct fragments—an N‐terminal fragment called “Nub” and a C‐terminal fragment called “Cub”. The Cub moiety is conjugated to an artificial transcription factor and then fused to a cytosolic terminus of a membrane‐bound protein (the “bait”). The Nub moiety is fused to potential interacting partners (“preys”), which can be either membrane‐associated or soluble. Interaction of bait and prey proteins brings the Nub and Cub moieties into close proximity, allowing them to form a “pseudoubiquitin” molecule, which is recognized by cellular deubiquitinating enzymes that cleave after the Cub C‐terminus. This releases the transcription factor, which then enters the nucleus and activates a reporter system (Stagljar *et al*, 1998; Snider *et al*, 2010).

#### Advantages

Membrane yeast two hybrid is simple, low cost, and scalable for use in both low‐ and high‐throughput (HT) formats. It is easy to establish in any laboratory environment and requires no specialized equipment. The assay is performed *in vivo* in a yeast host, allowing for the study of the interactions of membrane proteins in their full‐length form and in the proper context of a membrane environment. This is a significant advantage over the classical Y2H. MYTH is best suited for the detection of binary interactions(Paumi *et al*, 2007; Deribe *et al*, 2009; Snider *et al*, 2010, 2013).

#### Limitations

Membrane yeast two hybrid suffers from some of the same disadvantages as the classical Y2H, including the problems associated with the expression, modification, and interaction of non‐native proteins in a yeast host, and artifacts resulting from protein overexpression. Also, MYTH can only be used with membrane proteins that have at least one terminus in the cytosol (where the necessary deubiquitinating enzymes are located). Additionally, soluble proteins cannot be used as baits in the MYTH system, unless they are exceptionally large or anchored to intracellular structures (thereby preventing diffusion of the bait‐transcription factor into the nucleus and interaction‐independent activation of the reporter system). The readout of this method is also indirect, preventing spatial or temporal analysis of PPIs (Snider *et al*, 2010).

### Luminescence‐based mammalian interactome mapping (LUMIER)

#### Principle

The LUMIER assay (Fig 1C) is a co‐immunoprecipitation‐based approach. In this method, one protein (“A”) is fused to Renilla luciferase, while another protein (“B”) is linked to an affinity tag (e.g., FLAG, HA, protein A). Tagged constructs are transfected into appropriate cell lines where they are overexpressed. Cells are then lysed and protein “B” is immunoprecipitated using an appropriate antibody against the affinity tag. Interaction with protein “A” is assessed by measuring luciferase activity brought down with protein “B” (Barrios‐Rodiles *et al*, 2005; Blasche & Koegl, 2013).

#### Advantages

The LUMIER assay is easy to perform and can be used in a HT screening format. It does not require specialized equipment, beyond standard reagents for cell culture and instrumentation to measure bioluminescence. The approach can be used in different cell lines, providing the option of studying PPIs for a given organism in an appropriate *ex vivo* format. Note that this assay is well suited for studying binary interactions, although indirect interactions can also be detected (Barrios‐Rodiles *et al*, 2005; Blasche & Koegl, 2013; Taipale *et al*, 2014).

#### Limitations

A major disadvantage of the LUMIER method is that it requires lysis of cells prior to immunoprecipitation, a process that can result in the disruption of weak and transient PPIs, as well as the introduction of potential artifacts (e.g., by bringing together proteins in the lysate, which might not normally interact with one another in the cell, destabilizing proteins and exposing previously concealed non‐native binding surfaces). The LUMIER assay must be carefully controlled, to normalize for differences in transfection efficiency and expression, and minimize background signal. The assay is not ideal for studying how PPIs change spatially, over time or in response to different environmental conditions (Barrios‐Rodiles *et al*, 2005; Blasche & Koegl, 2013).

### Mammalian protein–protein interaction trap (MAPPIT)

#### Principle

The MAPPIT assay (Fig 1D) is designed for use in mammalian cell lines and is based on a cytokine signal transduction mechanism. A “bait” protein is fused to the C‐terminus of a cytokine receptor deficient in binding to STAT3 (required for signal transduction), while “prey” proteins are fused to receptor fragments containing functional STAT3 recruitment sites. An interaction between a bait and prey proteins produces a functionally competent receptor, which, in response to cytokine ligand stimulation, activates STAT3 molecules (through intermediate JAK kinase activity), allowing them to enter the nucleus and induce transcription of a reporter system (e.g., luciferase; Ulrichts *et al*, 2009).

#### Advantages

Mammalian protein–protein interaction trap provides a powerful way to examine mammalian PPIs directly in the context of the mammalian cell and is suitable for use in both HT library and array screening formats. The assay is easy to perform and does not require specialized equipment, beyond the necessary cell culture reagents and instrumentation to measure bioluminescence or fluorescence. Note that this method is best suited for studying binary interactions (Lievens *et al*, 2009, 2011). Variations of MAPPIT are effective for use in small‐molecule screening approaches (Eyckerman *et al*, 2005; Caligiuri *et al*, 2006; Lievens *et al*, 2011).

#### Limitations

Anchoring of the interaction sensor (i.e., the cytokine receptor) to the plasma membrane requires that PPIs occur in the cytoplasmic submembrane region, preventing detection of interaction with preys localized to other subcellular compartments. This anchoring (and the large size of the bait tag) may also block certain PPIs due to steric issues beyond those occurring in many other methodologies (Lievens *et al*, 2009). Finally, the method is also not compatible with full‐length transmembrane proteins and is not suitable for spatial or temporal analysis of PPIs.

### Kinase substrate sensor (KISS)

#### Principle

Kinase substrate sensor (Fig 1E) is a recently developed mammalian two‐hybrid approach designed to measure intracellular PPIs. In this assay, a “bait” protein is fused to the kinase domain of TYK2, while “preys” are coupled to a gp130 cytokine receptor fragment carrying TYK2 substrate motifs. Interaction of bait and prey results in phosphorylation of gp130 by TYK2, resulting in docking and activation of STAT3, which can then enter the nucleus and activate transcription of a STAT3‐dependent reporter system (e.g., luciferase; Lievens *et al*, 2014).

#### Advantages

Kinase substrate sensor allows assessment of PPIs directly in living mammalian cells and is sensitive enough to detect dynamic changes in response to physiological or pharmacological challenges. The method is effective for use with both membrane and cytosolic proteins and is best suited for measuring binary interactions (Lievens *et al*, 2014).

#### Limitations

Like many other assays, the KISS readout is indirect, preventing spatial or temporal analysis of PPIs. The assay relies on endogenous STAT3, making this approach unsuitable for studying interactions involving proteins or stimuli that affect STAT3 signaling (Lievens *et al*, 2014).

### Bimolecular fluorescence complementation (BiFC)

#### Principles

Bimolecular fluorescence complementation (Fig 1F) is based on the division of a fluorescent protein (e.g., YFP) into two distinct non‐fluorescent fragments, which are then fused to “bait” and “prey” proteins of interest. Interaction between bait and prey allows the two non‐fluorescent fragments to associate and form a fluorescent complex, which can be viewed by microscopy or flow cytometry (Kerppola, 2008; Zhang *et al*, 2015).

#### Advantages

Bimolecular fluorescence complementation allows direct visualization of PPIs in living cells, providing spatial information about the subcellular location where PPIs are occurring. The method is highly sensitive and can be used to detect interactions between proteins expressed at endogenous or near‐endogenous levels, as well as weak and transient interactions. The method can be used for different organisms, is simple to set up, and is cost‐effective. Different fluorescent proteins can also be used in combination, allowing the visualization of multiple PPIs in parallel in single cells. The method is best suited for detecting binary interactions (Hu *et al*, 2002; Kerppola, 2008; Zhang *et al*, 2015).

#### Limitations

Bimolecular fluorescence complementation is not ideal for measuring PPI dynamics or real‐time changes, due to a delay in generation of fluorescence upon protein interaction, as well as the irreversible nature of fluorochrome formation (Kerppola, 2008). Another disadvantage of BiFC includes functionality of fusion proteins, as is the case for other techniques involving protein tagging. Lastly, in some cases false‐positive fluorescent signals can be detected by BiFC due to fluorescence intensity of reconstituted fragments arising irrespective of (or from non‐specific) interaction between two proteins under investigation (Miller *et al*, 2015).

### Mammalian membrane two hybrid (MaMTH)

#### Principle

Mammalian membrane two hybrid (Fig 1B) is a recently developed *in vivo* proteomics technology designed for the analysis of mammalian membrane PPIs. The assay is based on the principle of split‐ubiquitin, wherein reconstitution of inactive fragments of ubiquitin (Nub and Cub) upon interaction of proteins to which they are fused leads to release of an artificial transcription factor, and subsequent expression of a reporter system (luciferase in the case of MaMTH; Petschnigg *et al*, 2014).

#### Advantages

Mammalian membrane two hybrid allows the analysis of the interactions of full‐length mammalian membrane proteins directly in their natural cellular context. The assay is low cost, highly scalable, and readily transferable to virtually any cell line of interest. No specialized equipment is required, beyond standard cell culture reagents and tools necessary for monitoring luciferase activity. One of the key advantages of MaMTH is its high sensitivity, making it suitable for both the measurement of weak/transient interactions, and for monitoring dynamic, “condition‐dependent” PPIs (i.e., which change in response to agonist, phosphorylation state, mutation etc.). The method is best suited for the detection of binary PPIs (Petschnigg *et al*, 2014).

#### Limitations

For MaMTH to function, the bait must be associated with the membrane or other intracellular structures, to prevent non‐specific activation of the reporter system (note that like MYTH, preys can be either soluble or membrane‐bound). Additionally, the termini of the membrane protein fused to Cub must be cytosolic, in order to provide access to the deubiquitinating proteases responsible for cleavage and release of transcription factor. The method is also not suitable for spatial or real‐time temporal analysis of PPIs (Petschnigg *et al*, 2014).

### Fluorescence resonance energy transfer (FRET)

#### Principle

Fluorescence resonance energy transfer (Fig 1G) is based on the non‐radiative transfer of energy from an excited donor fluorophore to a nearby acceptor molecule. Donor and acceptors are selected such that the absorption spectrum of the acceptor fluorophore overlaps with the emission spectrum of the donor. In this approach, one protein of interest is fused to the donor, while the other is fused to the acceptor. If the two proteins interact or come into close proximity with one other, the donor and acceptor fluorophores are also brought together. Excitation of the donor in this case does not lead to photon release, but rather energy transfer to the nearby acceptor, which in turn produces an emission signal. This emission signal is distinct from the signal that would be observed for donor alone, and is used to monitor PPI (Ma *et al*, 2014).

#### Advantages

A major advantage of FRET is its ability to monitor instantaneous, real‐time PPIs, allowing the measurement of short‐lived transient interactions. In addition, FRET can be used directly in the context of live cells and allows detection of interaction sites. Also, due to the reversible nature of the fluorophore interaction, complex interaction dynamics can be monitored such as the dynamic equilibrium between complex formation and dissociation (Ma *et al*, 2014).

#### Limitations

For FRET to function, protein fusions to appropriate fluorophores need to be generated (the technical demands of which may vary depending upon the fluorophores selected). In addition, for a strong FRET readout, close spatial proximity of the fluorophores is required for the energy transfer to occur. FRET also has decreased sensitivity compared to other fluorescence‐based approaches like BiFC or BRET, as there tends to be strong background autofluorescence in cells upon sample illumination. For this reason, many controls are necessary to quantify the changes in fluorescence intensity in the presence and absence of energy transfer, and particularly weak interactions producing a signal close to background may be difficult to detect. Depending upon the fluorophores selected, photobleaching can also result in loss of signal over time (Boute *et al*, 2002; Ma *et al*, 2014).

### Bioluminescence resonance energy transfer (BRET)

#### Principle

The BRET assay (Fig 1G) has been developed to diminish a major limitation of FRET—the strong background signal that results from the direct excitation between the donor and acceptor fluorophores. In BRET, a protein of interest is fused to *Renilla* luciferase (“RLuc”, serving as the energy donor), while its interacting partner is fused to either green or yellow fluorescent protein (GFP or YFP, serving as the energy acceptor). When donors and acceptors are brought into close proximity (< 100 Å) by interaction of their fusion partners, energy transfer occurs, producing fluorescent signal which is monitored to detect the PPIs (Boute *et al*, 2002; Hamdan *et al*, 2006).

#### Advantages

Like FRET, BRET is able to monitor instantaneous real‐time PPIs, functions directly in the context of live cells, and provides information about the cellular location at which an interaction occurs (Boute *et al*, 2002; Hamdan *et al*, 2006; Xie *et al*, 2011). However, BRET also has greater sensitivity than FRET, with lower background (Boute *et al*, 2002).

#### Limitations

The major limitations of BRET are similar to those of FRET, including the need for the generation of fusion proteins, and the efficiency of the assay being dependent on close spatial proximity of the donor and acceptor (in order for proper energy transfer to occur; Hamdan *et al*, 2006). BRET signal also tends to be significantly weaker than that produced by FRET (Hamdan *et al*, 2006; Xie *et al*, 2011). In addition, the analysis of PPIs using BRET and FRET is not as easily scalable to HT screening applications as other methods, making it better suited to screens involving a more limited number of potential hits.

### Affinity purification–mass spectrometry (AP‐MS)

#### Principle

Affinity purification–mass spectrometry (Fig 1H) is a popular technology that has gained considerable attention over the past decade. The general principle involves immobilization of “bait” protein of interest on a solid support (most frequently agarose or magnetic beads), and use of this coupled “bait” to capture target protein(s) from a soluble phase. Once affinity‐purified, captured proteins are usually digested with proteases (e.g., trypsin), to generate peptides, which in turn are sub‐fractionated using high‐pressure liquid chromatography (HPLC) and then ionized and detected using a mass spectrometer. AP‐MS can be conducted either with endogenous, native protein baits (using specific antibodies raised against them) or with protein baits to which a standardized “epitope tag” (e.g., TAP‐, FLAG‐, c‐myc‐, HA‐, His‐, protein A‐, Strep‐Tag) is fused. The choice of the most appropriate affinity purification method depends on a combination of factors, including the availability of antibodies, the type of a protein under investigation, and the scale of the conducted analysis (Dunham *et al*, 2012).

#### Advantages

Affinity purification–mass spectrometry is a library‐independent method with true genomewide HT capability. The main advantage of affinity purification using native antibodies against endogenous, native “baits” is that the proteins are purified in their natural form from cell or tissue lysates, eliminating issues associated with protein tagging and allowing multiple isoforms to be interrogated simultaneously. Conversely, the main advantages of epitope tagging are that it allows the study of proteins for which native antibodies are not available, and the analysis of multiple proteins using a single, defined process with a specific antibody (i.e., since many different proteins can be tagged with a single epitope; Dunham *et al*, 2012).

#### Limitations

The major limitation of any AP‐MS approach is a need to perform cell lysis and affinity purification. These steps do not allow for the detection of spatial or temporal PPIs and can also prevent detection of weak, transient PPIs. Another major limitation is contamination by abundant proteins co‐purified from AP (Dunham *et al*, 2012) and artifacts resulting from exposure of proteins to one another in the unnatural environment of a cellular lysate (e.g., spurious interactions, disruption of protein interactions). In cases where epitope tags and ectopic expression are required, high background can also result from improper folding and mislocalization. However, several strategies do exist, which may help overcome these problems, such as use of appropriate negative controls, further enrichment of true interactions using tandem affinity purification (TAP), quantification approaches (SILAC and other isotopic labeling, and label‐free quantification, which usually need special computational tools; Choi *et al*, 2011) and, for smaller experiments, contaminants can be filtered out by comparison with a contaminant repository database (Mellacheruvu *et al*, 2013). With endogenous proteins, low expression levels of proteins of interest may also prevent detection (Dunham *et al*, 2012). Lastly, data analysis of AP‐MS experiments is more difficult compared to other PPI assays (i.e., Y2H), due to required expertise with MS and specific bioinformatics tools needed to address the limitations listed above.

### Proximity‐dependent biotin identification coupled to mass spectrometry (BioID‐MS)

#### Principle

Proximity‐dependent biotin identification coupled to mass spectrometry (Fig 1I), which is similar in nature to AP‐MS, uses a “bait” protein of interest fused to a prokaryotic biotin ligase molecule (BirA). When expressed in cells, proteins in proximity to the BioID fusion protein are biotinylated by BirA, permitting their selective isolation using an avidin/streptavidin‐based biotin affinity capture approach. These purified, biotinylated proteins are then identified using MS, providing a list of candidate interacting partners for the bait of interest (Roux *et al*, 2012).

#### Advantages

Similar to AP‐MS, BioID‐MS is also a library‐independent method. A major advantage of BioID‐MS is that PPIs are detected in their natural cellular context (since biotinylation occurs in the cell prior to lysis). Additionally, issues associated with bait/prey stability and disruption of interactions upon cell lysis are avoided. The method is also well suited for identifying weak or transient interactions and is amenable to temporal regulation (potentially allowing for pulse‐chase type applications; Roux *et al*, 2012). The method also appears to be more effective at detecting low‐abundance proteins than AP‐MS (Lambert *et al*, 2015).

#### Limitations

BioID‐MS requires the fusion of bait protein to BirA, which adds significantly to the size of the protein and can potentially compromise its targeting or function. Low expression level of PPI partners can also lead to false negatives. The biotinylation process itself may also affect protein behavior/interactions in certain cases (Roux *et al*, 2012). Finally, like AP‐MS, required expertise in MS and specific bioinformatics tools can make data analysis more complex than for other methods.

### Proximity ligation assay (PLA)

#### Principle

Proximity ligation assay (Fig 1J) is a powerful method for the *in situ* detection of PPIs in fixed cells and tissues. The general premise of the assay involves the use of proximity probes (i.e., antibodies conjugated with DNA oligonucleotides, which are able to recognize two target proteins of interest). When the Proximity Probes are brought close to one another (i.e., due to interaction of the target proteins to which they are bound), the DNA strands serve as a template to direct ligation of two subsequently added oligonucleotide fragments into a circular molecule. This circular DNA is then amplified using rolling circle amplification (RCA) primed by one of the original proximity probe oligonucleotides, resulting in a long DNA sequence physically linked to the corresponding antibody (and thus the interacting protein pair). This new DNA sequence contains many repetitive elements, which are then bound by fluorophore‐labeled complementary oligonucleotide probes, allowing visualization of interactions, at the specific sites where they occur, using a fluorescence microscope (Koos *et al*, 2014).

#### Advantages

The major advantages of PLA are its ability to detect and localize PPIs with single molecule resolution and objectively quantify them in cells and tissues. In addition, transient or weak interactions can be monitored (Koos *et al*, 2014).

#### Limitations

A major disadvantage of PLA is the dependence on enzymes (i.e., for ligation and polymerization), making the approach expensive and highly dependent on enzyme activity and stability. The required use of antibodies in PLA is another potential drawback, as antibodies are often costly, and may also not be readily available against particular proteins of interest (Koos *et al*, 2014). Thus, PLA is not ideally suited for HT PPI screening applications.

### Ligand–receptor capture – trifunctional chemoproteomics reagents (LRC‐TriCEPS)

#### Principle

The LRC‐TriCEPS approach (Fig 1K) has been developed to elucidate potential receptor/ligand interactions. TriCEPS employs a chemoproteomics reagent consisting of three moieties—one that binds ligands of interest containing an amino group, a second that binds glycosylated receptors on live cells, and a biotin tag. The reagent effectively serves as a stable “bridge”, covalently linking a ligand of interest to carbohydrate groups on its cognate receptor. Following treatment with TriCEPS, cells are lysed and enzymatically digested with trypsin, and TriCEPS bound peptides are purified via the biotin tag. Receptor peptides are then freed from the TriCEPS reagent and identified using quantitative MS (Frei *et al*, 2012, 2013).

#### Advantages

The major advantages of using LRC‐TriCEPS are the ability to detect ligand–receptor interactions without the need for genetic manipulations. This approach is also effective for detecting surface interactions that are very transient in nature, and can be used with both populations of individual cells and tissue samples. In addition, LRC‐TriCEPS can be used to identify the cell surface binding partners of many different types of ligands, including peptides, proteins, viral particles, antibodies, and engineered affinity binders (Frei *et al*, 2012, 2013).

#### Limitations

By design, LRC‐TriCEPS is only useful for identifying N‐glycoprotein receptors, and is ineffective if glycans are sterically inaccessible. Coupling of ligands to TriCEPS reagent may also affect their functionality/proper target binding in some cases (necessitating verification of ligand function following TriCEPS linkage, where possible). TriCEPS may also not be effective in detecting receptor–ligand interactions in situations where ligand binding requires association to other cell surface structures (in addition to a target glycoprotein; Frei *et al*, 2013).

### Avidity‐based extracellular interaction screen (AVEXIS)

#### Principle

Avidity‐based extracellular interaction screen is a PPI assay developed to systematically screen for novel extracellular receptor–ligand pairs involved in cellular recognition processes (Fig 1L). The general premise of this approach involves the expression of secreted recombinant “bait” and “prey” proteins (e.g., natively secreted proteins or the truncated ectodomain of membrane proteins containing an N‐terminal secretory peptide) in a mammalian cell‐based system so that structurally important posttranslational modifications can occur. Bait proteins are biotinylated, so they can be captured on a streptavidin‐coated solid phase, while prey proteins are tagged with β‐lactamase and a peptide sequence directing their pentamerization (used to increase effective prey concentration and improve assay sensitivity). Bait and prey isolates are then presented to one another in a binary manner to detect direct interactions using an ELISA‐type format (Kerr & Wright, 2012).

#### Advantages

Avidity‐based extracellular interaction screen can detect very weak PPIs, which are a typical feature of interactions between membrane‐embedded receptor proteins (it has been shown to detect interactions with equilibrium dissociation constants as low as ~10 μM) with a low false‐positive rate (Sun *et al*, 2012; Kerr & Wright, 2012). The assay has also been adapted for use on a higher‐throughput scale than many other assays designed to detect extracellular interactions (Sun *et al*, 2012).

#### Limitations

Avidity‐based extracellular interaction screen is limited to the study of membrane proteins with self‐contained extracellular domains and is not generally suitable for multi‐pass membrane proteins and other proteins that need to be embedded in the plasma membrane to fold and function properly (Kerr & Wright, 2012; Frei *et al*, 2013). In addition, selecting, preparing, and validating the constructs necessary for AVEXIS can be a lengthy (and relatively costly) process, although the use of a recently reported protein microarray format does help in this regard (Sun *et al*, 2012). The approach may also have difficulty detecting homophilic interactions and is not ideal for quantitatively comparing the strength of different interactions (due to the artificial pentamerization of preys; Kerr & Wright, 2012).

## Dynamic protein interaction networks

A major limitation of the available PPI interaction network data is the static representation of these interactions, neglecting the temporal and spatial organization of protein dynamics as well as the effect of posttranslational modifications (PTMs). For instance, a PPI may occur only during specific time periods (e.g., under particular stress conditions, in response to certain signaling events etc.) or if specific PTMs are present. The nature of protein–protein interaction is thus an inherently dynamic process that changes with time, environments, and at different stages of the cell cycle. Recently, dynamic protein interaction networks have been constructed by using proteomic, genomic, and transcriptomic methodologies. In the previous section, we touched briefly on the suitability of some techniques for mapping dynamic interactions. Examples of some of specific proteomic‐based approaches employed include Y2H and AP‐mass spectrometry (Woodsmith & Stelzl, 2014). For instance, the phosphotyrosine‐dependent PPI network was recently studied using Y2H, and identified many novel phosphotyrosine‐dependent PPIs of human kinases (Grossmann *et al*, 2015). AP‐MS approaches have been employed to study the dynamics of the human 26S proteasome‐interacting proteins (Wang & Huang, 2008, 2014), study changes in the interactome of 14‐3‐3β in response to activation of the insulin‐PI3K‐AKT pathway (Collins *et al*, 2013), and map phosphotyrosine‐dependent interaction sites on ErbB‐receptor family members (Schulze *et al*, 2005).

In addition to the temporal and spatial organization of PPIs, perturbations of PPIs from disease‐associated alleles have also gained much interest. Such perturbations can be either subtle or dramatic, but often have significant biological consequences, and understanding the nature of these changes can be important in developing new therapeutic strategies. Thousands of genetic variants have been identified in many Mendelian disorders, complex traits, and cancers; however, the effect of these genetic variants on PPI networks is still far from clear. Recent studies have looked into assessing perturbations of protein interactions by disease‐associated alleles using the techniques described above. For example, Sahni *et al* (2015) found widespread perturbations of macromolecular interactions caused by disease‐specific mutant alleles using a comprehensive genomics/proteomics approach involving LUMIER and Y1H/Y2H technologies. Additionally, Wang *et al* (2012) integrated available protein structure and large‐scale PPI data to comprehensively investigate the relationships between mutations, protein interactions, and human disease. Work by Petschnigg *et al* (2014) using the MaMTH assay also identified differential interactors of WT and oncogenic mutant forms of the receptor tyrosine kinase EGFR. For a more thorough examination of protein interactome networks and disease, several excellent reviews are available (Ideker & Sharan, 2008; Vidal *et al*, 2011; Sahni *et al*, 2013).

## Analysis of PPI screen data

Once a screen is completed, it is necessary to properly analyze the data in order to validate the results and improve overall interactome quality. In this section, we provide an overview of some key considerations and methods useful for analysis of PPI datasets.

### Assessing PPI datasets

Assessing the frequency of false positives and false negatives in PPI datasets has been a long‐standing problem, especially for HT screens. Typically, the frequency of false positives is measured as the false discovery rate ($FDR=\frac{false\;positives}{true\;positives+false\;positives}),$ and the frequency of false negatives as the false negative rate ($FNR=\frac{false\;negatives}{true\;positives+false\;negatives}$) or sensitivity ($\frac{true\;positives}{true\;positives+false\;negatives}$). The main strategies for assessing FDR and sensitivity have involved testing detected interactions by multiple methods and comparing against interactions from literature. FDR has been, arguably, a greater focus in interactome studies than sensitivity. In small‐scale screens, FDR can be assessed and minimized by testing all reported interactions using multiple methods. However, the FDR of small‐scale screens may still be uncertain. Edwards *et al* (2002) found that interactions from small‐scale screens were not always consistent with known 3D structures of protein complexes. Rolland *et al* (2014) tested interactions reported in single publications and found that the detection rate was just slightly higher than for random protein pairs. Small‐scale studies rarely report protein pairs that were tested but not detected. Consequently, the sensitivity of their screens and the assessment of how much of the interactome they have tested are largely unknown (Cusick *et al*, 2009).

Assessing HT screens is more difficult since testing all detected interactions by multiple methods is not feasible. Venkatesan *et al* (2009) developed a rigorous framework for assessing the quality of HT PPI datasets. Their framework calculates four parameters: screening completeness, assay sensitivity, sampling sensitivity, and precision. Screening completeness is the fraction of open reading frame (ORF) pairs tested in the screen. Assay sensitivity is the fraction of interactions that can be identified by the assay, estimated by testing the assay on a gold standard set of interactions, and determining the fraction detected. Sampling sensitivity is the fraction of detectable interactions identified in one trial of the assay, estimated by repeating the assay multiple times and fitting a Bayesian model to the results. Precision, the fraction of detected pairs that are true positives, can be estimated by testing the assay on reference sets of interacting and non‐interacting protein pairs, and calculating the fraction of detected pairs that are from the interacting set.

Once the precision and sensitivity of an assay have been estimated, the assay can be used to determine the FDR of interaction datasets. HT studies commonly estimate FDR by retesting a subset of detected protein pairs using different small‐scale or HT methods (Yu *et al*, 2008; Simonis *et al*, 2009; Rolland *et al*, 2014). Such estimates need to take into account the precision and sensitivity of the retesting assay. This is especially important as the precision or sensitivity may be quite limited. Braun *et al* (2009) assessed five HT assays on a gold standard dataset comprised of interactions reported by multiple small‐scale studies (positive cases) and an equal number of randomly chosen protein pairs. Each HT assay detected only ~20–35% of positive cases, and up to 4% of negative cases. Combined, the five assays detected 59% of positive cases, while FDR increased to 14%. Furthermore, different assays have different systematic biases; for example, affinity purification methods may be biased in favor of high‐abundance proteins (Ivanic *et al*, 2009). Another concern, especially difficult to address, is that gold standard datasets may also have biases. Such datasets, often comprising PPIs reported by multiple small‐scale studies, may be deficient for certain types of proteins or PPIs due to research bias or limitations of assays (Hakes *et al*, 2008; Edwards *et al*, 2011; Rolland *et al*, 2014; Kotlyar *et al*, 2015; Wang *et al*, 2015).

### Computational methods for assessing results

Computational methods provide a means of estimating FDR without retesting detected protein pairs. Furthermore, they can provide error estimates for specific proteins or protein pairs.

D'haeseleer and Church (2004) introduced a method for estimating FDR and assessing the reliability of individual interactions. Their method analyzes the overlap of detected interactions with two other datasets, including a trusted reference set. The variance of FDR estimates can be high if the overlaps are small. However, the authors showed that FDR estimates are not greatly affected by the quality of the chosen datasets.

A statistical model introduced by Huang and Bader for assessing two‐hybrid datasets provides global error estimates as well as error estimates for specific baits (Huang & Bader, 2009). Thus, it can determine whether certain baits are responsible for a disproportionate share of the global error rate. For example, in two‐hybrid data from worm, it found that the FDR was especially high among proteins involved in cellular metabolic processes.

### Computational methods to help improve data quality

One approach for assessing and improving data quality is to examine whether a PPI dataset possesses properties of interacting protein pairs. Methods that use this approach assume that interacting proteins are likely to have co‐expressed genes (Deane *et al*, 2002), shared subcellular localization (Sprinzak *et al*, 2003), similar functional and process annotations (Sprinzak *et al*, 2003; Wang *et al*, 2007), and shared interaction partners (Saito *et al*, 2002; Goldberg & Roth, 2003). Evaluating a PPI dataset using such evidence can be problematic: Many interacting protein pairs do not have correlated gene expression, protein annotations such as subcellular localization and function are often incomplete or unavailable, and shared interaction partners are frequently unknown, since the interactomes of most species are largely unmapped. However, ranking detected protein pairs using these types of evidence can help identify true positive interactions. A combination of such evidence has been used in HT studies to define high‐confidence (HC) subsets of detected interactions (Miller *et al*, 2005; Havugimana *et al*, 2012). The evidence can also help identify potential false negatives—protein pairs that are not strongly supported by the experimental detection method but have properties of true interactions. Unfortunately, ranking based on this evidence can introduce biases; ranking by correlation of gene expression profiles favors stable interactions (Brown & Jurisica, 2007), while ranking by shared Gene Ontology terms or interaction partners favors well‐studied proteins. If the evidence is used multiple times during the planning and analysis of an experiment, there may be a danger of circular reasoning. For example, testing protein pairs with similar functions and then ranking detected pairs by similarity of localizations would be largely ineffective, as functional similarity is correlated with localization similarity.

An approach for improving the quality of AP‐MS data involves calculating a score for each co‐purified pair, indicating the likelihood of the two proteins being observed together. Such scores have been calculated using various methods: log‐ratios of observed versus expected co‐occurrences (Gavin *et al*, 2006), machine‐learning algorithms (Krogan *et al*, 2006; Collins *et al*, 2007), hypergeometric probabilities (Hart *et al*, 2007), and randomizations (Yu *et al*, 2009). Scores can be used for ranking protein pairs and defining a HC subset of interactions. A score threshold for defining this subset can be determined based on FDR and sensitivity calculated from a gold standard dataset comprising interacting and non‐interacting protein pairs. True positive interactions can be distinguished from contaminants by analyzing quantitative information from mass spectrometry data, including spectral counts, signal intensity in the precursor scan of the mass spectrometer, and intensity of product ions after fragmentation (Gingras & Raught, 2012). Analysis of quantitative data using tools such as SAINT (Choi *et al*, 2011) can be especially helpful when aiming to detect transient interactions; preservation of transient interactions in AP‐MS experiments requires short incubation times and few washes, resulting in more contaminants that need to be filtered (Gingras & Raught, 2012).

Varjosalo *et al* (2013) filtered AP‐MS data in three steps to remove different types of protein contamination. The first filter removed proteins that may have been left over from a proceeding experiment. The second filter removed non‐specifically interacting proteins. The third filter removed low‐abundance, non‐systematic contaminants; bait–prey pairs were assigned weighted spectral‐count‐based scores reflecting interaction abundance and reproducibility, and pairs with scores below a threshold were removed. This three‐step filtering improved reproducibility of resulting networks more than previous filtering methods, wD‐score (Behrends *et al*, 2010) and SAINT (Choi *et al*, 2011).

### Computational prediction to help improve datasets and the interactome

Computational PPI prediction is similar to previously described methods that assign scores to detected protein pairs, indicating the likelihood of interaction. However, prediction methods provide scores for both detected and undetected PPIs. These scores can be used to improve the quality of experimentally detected PPIs, by identifying high‐confidence subsets and potential false negatives, which can help accelerate interactome mapping (Schwartz *et al*, 2009). Also, prediction methods can help fill the gaps in a known interactome by predicting interactions for interactome orphans and low‐degree proteins (Kotlyar *et al*, 2015).

PPI prediction methods can be categorized by the data they use: genomic data, protein sequence, protein structure, PPI networks, gene expression, and annotations of gene function, localization, and process. Prediction methods based on genomic data analyze conserved operon structure, fusion domains, phylogenetic profiles, and interologs. Analysis of operon structure is based on the idea that genes in close proximity on the genome are more likely to encode interacting proteins, especially if the proximal locations are conserved across species (Dandekar *et al*, 1998; Overbeek *et al*, 1999). Similarly, if two genes exist as a single fused gene in another species, they are likely to encode interacting proteins (Enright *et al*, 1999). Phylogenetic profiles are used to identify gene pairs that tend to co‐occur across species—either both are present in a species, or both are absent; such pairs are likely to be functionally related and may encode interacting proteins (Pellegrini *et al*, 1999). Interologs are interactions conserved across species: if a pair of proteins interacts in one species, their orthologs in another species are more likely to interact (Walhout *et al*, 2000; Yu *et al*, 2004).

Many studies have predicted interactions based on protein sequence (Gomez *et al*, 2003; Martin *et al*, 2005; Nanni & Lumini, 2006; Shen *et al*, 2007; Guo *et al*, 2008; Zaki *et al*, 2009; Chang *et al*, 2010; Guo *et al*, 2010; Yu *et al*, 2010). These studies analyze experimentally determined interactions to find patterns that distinguish sequences of an interacting protein pair from those of a non‐interacting pair. This is often done using machine‐learning algorithms such as support vector machines, random forests (Roy *et al*, 2009), and K‐local hyperplane nearest‐neighbors (Nanni & Lumini, 2006). Protein sequence can also be used indirectly for prediction; protein domains can be determined from sequence, and pairs of domains enriched among known interacting protein pairs may predict new interactions (Sprinzak & Margalit, 2001; Wojcik & Schächter, 2001; Nguyen & Ho, 2006; Singhal & Resat, 2007). Another approach combines sequence with protein tertiary structure. Although few proteins or complexes have known 3D structure, sequence homology can serve as a link to other proteins. Homologous proteins, especially with conserved binding sites, are likely to interact in similar ways (Aloy & Russell, 2003; Ma *et al*, 2003; Sinha *et al*, 2010). Thus, a protein pair can be predicted to interact based on sequence or structural homology to proteins in solved complexes (Lu *et al*, 2002; Aloy & Russell, 2003; Zhang *et al*, 2012a).

If an interactome is partially known, new interactions may be predicted from known network structure, often based on the idea that interacting proteins tend to share interaction partners (Saito *et al*, 2002; Goldberg & Roth, 2003; Liu *et al*, 2008). Other types of interaction evidence—including correlated gene expression, shared subcellular localization, similar function and process—are typically used in combination with other evidence (Jansen *et al*, 2003; Ben‐Hur & Noble, 2005; Rhodes *et al*, 2005; Elefsinioti *et al*, 2011).

PPI prediction methods have a number of limitations and biases, often similar to those of experimental PPI assays. Computational methods tend to have difficulty predicting transient interactions and interactions involving lesser studied proteins, which typically have no tertiary structure data, no detailed Gene Ontology or domain annotations, few known interactions, and few orthologs in different species (Kotlyar *et al*, 2015). Transient interactions are difficult to predict based on correlation of gene expression profiles (Brown & Jurisica, 2007), or analysis of protein sequence or structure. In these interactions, the two encoding genes are not highly correlated (Brown & Jurisica, 2007), interaction interface sequences are not as conserved as in obligate interactions (Perkins *et al*, 2010), interacting proteins often undergo conformational changes (Perkins *et al*, 2010), and interactions are frequently mediated by linear motifs rather than globular domains (Perkins *et al*, 2010). If proteins lack Gene Ontology annotations, known interaction partners, or orthologs, it is difficult to predict their interactions using annotation similarity, network topology, or comparative genomics, respectively. Interestingly, such proteins are also underrepresented in the experimentally detected human interactome (Kotlyar *et al*, 2015). If prediction methods require training, they may acquire the biases of their experimentally detected training set. This may explain the finding of Rolland *et al* that a proteome‐wide, structure‐focused prediction method, PrePPI (Zhang *et al*, 2012a), had a tendency to report interactions among well‐studied proteins (Rolland *et al*, 2014).

## Databases: what is available and what do they tell us?

Studies that detect PPIs report their findings in journal articles as free‐form text. Consequently, original information about detected PPIs is scattered across thousands of articles and requires manual curation. Converting these data into an easily usable set of interactions and experimental descriptions remains a daunting problem, comprising several key tasks: (i) experiments need to be described with a controlled vocabulary and recorded in a common format, (ii) thousands of articles have to be curated and resulting information has to be easily accessible, and (iii) proteins need to be unambiguously identified. The first task, creation of standard vocabularies and formats for PPI data, was addressed through the Human Proteome Organization Proteomics Standards Initiative (HUPO‐PSI) by the Molecular Interaction (MI) workgroup. They created a common controlled vocabulary for experimental techniques, molecular features, and interaction types (Orchard & Kerrien, 2010), and XML (PSI‐MI XML) and tab‐delimited (MITAB) formats for recording and transferring data (Kerrien *et al*, 2007). Most major PPI databases adopted the vocabulary and data formats, allowing users to easily integrate datasets and analyze them with programs such as Cytoscape (Su *et al*, 2014) and NAViGaTOR (Brown *et al*, 2009).

**Figure 2:**
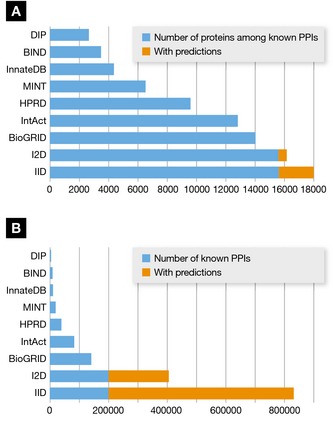
Protein and PPI counts in major human PPI databases (A) Major human PPI databases and the number of proteins they contain. (B) Major human PPI databases and the number of PPIs they contain.

The second task, curating articles and providing results through online databases, started with the DIP (Salwinski *et al*, 2004) and BIND (Bader *et al*, 2003) database projects and has continued with the creation of many similar resources (Table 2 and Fig 2A). These resources were especially important given the rapid increase in the number of human PPIs (Fig 2B) detected by various experimental methods. Initially, the focus was on experimentally detected PPIs in yeast and human, but the scope has greatly expanded. Some databases now include computationally predicted interactions (e.g., STRING (Szklarczyk *et al*, 2015), FpClass (Kotlyar *et al*, 2015), IID (Kotlyar *et al*, 2016)), functionally related protein pairs (e.g., STRING (Szklarczyk *et al*, 2015)), interactions between proteins and other molecule types (e.g., BIND (Bader *et al*, 2003), BindingDB (Liu *et al*, 2007), IntAct (Kerrien *et al*, 2012)), interactions in a range of organisms (e.g., DIP (Salwinski *et al*, 2004), IntAct (Kerrien *et al*, 2012), MINT (Licata *et al*, 2012), BioGRID (Chatr‐Aryamontri *et al*, 2015), IID (Kotlyar *et al*, 2016)), and interactions involving specific types of proteins (e.g., extracellular matrix—MatrixDB (Chautard *et al*, 2011), immune related—InnateDB (Breuer *et al*, 2013)).

**Table 2:** Major protein–protein interactions (PPI) databases

Database	Reference	URL	IMEx member	PPI evidence	Specialization
BioGRID	Chatr‐Aryamontri *et al* (2015)	http://thebiogrid.org	Observer	Experimental	
DIP	Salwinski *et al* (2004)	http://dip.doe-mbi.ucla.edu/dip	Yes	Experimental	
FPCLASS	Kotlyar *et al* (2015)	http://ophid.utoronto.ca/fpclass	No	Computational	
HPRD	Keshava Prasad *et al* (2009)	http://www.hprd.org/	No	Experimental	
IID	Kotlyar *et al* (2016)	http://ophid.utoronto.ca/iid	Yes	Computational, Experimental	
InnateDB	Breuer *et al* (2013)	http://www.innatedb.ca	Yes	Experimental	Immune‐related PPIs
IntAct	Kerrien *et al* (2012)	http://www.ebi.ac.uk/intact	Yes	Experimental	
iRefWeb	Turinsky *et al* (2014)	http://wodaklab.org/iRefWeb/	No	Experimental	
MatrixDB	Chautard *et al* (2011)	http://matrixdb.ibcp.fr/	Yes	Experimental	Extracellular matrix PPIs
MINT	Licata *et al* (2012)	http://mint.bio.uniroma2.it/mint	Yes	Experimental	
STRING	Szklarczyk *et al* (2015)	http://string-db.org	No	Computational, Experimental	Functional protein–protein associations

Databases also differ in several other respects: the level of detail recorded about experiments (shallow versus deep), the way information is acquired (manual curation of literature or automatic approaches such as text mining or PPI prediction), and the sources of information—peer‐reviewed articles (primary sources) or other databases (secondary sources). Manual curation of articles is the most trusted method for acquiring data and is carried out by most databases. Several approaches have been used to assist with curation: curation guidelines have been established, an automated syntax checker was implemented to test for compliance with accepted formats, and guidelines were created for reporting PPIs in papers, so that curation of papers is easier and more accurate (Orchard *et al*, 2007). However, manually curating all previously published literature and keeping up with new publications is a huge task. The IMEx consortium (Orchard *et al*, 2012) was created to better organize the curation effort across major PPI databases. Members of the consortium avoid curating the same papers and follow the same curation rules.

Although current PPI databases provide easy access to their PPI data, obtaining the most complete up‐to‐date network can be challenging: the latest data has to be downloaded from multiple databases and merged. The PSICQUIC (Aranda *et al*, 2011) query interface simplifies these tasks. It enables multiple PPI databases to be searched with the same query, and a clustering algorithm provided with PSICQUIC helps merge results by grouping interaction evidence based on primary identifiers.

Unfortunately, the third task, unambiguously identifying proteins, has not been entirely resolved. Most PPI databases use UniProtKB (Magrane & Consortium, 2011) protein identifiers, which can represent peptides, fusion proteins, specific isoforms, or proteins whose isoforms are not specified. However, some databases use Ensembl (Flicek *et al*, 2014), Entrez (Maglott *et al*, 2011), RefSeq (Pruitt *et al*, 2012), and species‐specific identifiers. Mapping between different types of identifiers is not always possible. When databases use different protein identifiers, PSICQUIC is unable to identify redundant data. However, this problem is being addressed as more providers of PSICQUIC clients include commonly used identifiers in export files (Orchard, 2012).

## Visualization, analysis, and biological validation of PPI data

### Visualization Tools

A number of tools are available for visualization and analysis of PPI data including Cytoscape (Su *et al*, 2014), NAViGaTOR (Brown *et al*, 2009), and packages from R and Bioconductor (e.g., Rintact (Chiang *et al*, 2008) combined with RBGL). The main types of functionality supported by these tools include loading PSI‐MI files, visualizing networks, annotating networks, and conducting network analysis. Data can be loaded from tab‐delimited or PSI‐MI XML files, and visualized as a graph, with nodes representing proteins and edges representing interactions. Multiple graph layouts are supported, including grids and force‐directed layouts. Annotations for nodes and edges can be included in the original data files, retrieved from other text files, or imported from databases. The appearance of nodes and edges can be set based on annotations. Network analysis capabilities include clustering to identify protein complexes, motifs, or graphlets, calculating centrality measures, and identifying shortest paths or flows.

### Identifying complexes in PPI networks

Although PPI networks focus on pairwise interactions, cellular processes are often carried out by protein complexes. Complexes typically have a “core”—a central functional unit present in most isoforms of the complex, and “attachments”—proteins present in some isoforms of the complex (Gavin *et al*, 2006). The attachments may include “modules”—sets of proteins that always appear together in different complexes (Gavin *et al*, 2006).

Experimental methods for detecting PPIs cannot easily identify complexes. For example, Y2H methods only identify binary interactions, and while TAP and HMS‐PCI identify potential complexes, reliably identifying complex members requires “reverse purification”—repeatedly applying the detection method, using candidate members of the complex as baits (Gavin *et al*, 2002).

Computational methods can predict complexes by analyzing PPI networks and integrating networks with information such as gene function or co‐expression. Most complex prediction methods share the same main steps: (i) assigning confidence scores to detected interactions, (ii) identifying complexes by clustering PPI networks or analyzing additional data, and (iii) evaluating resulting complexes by comparing with gold standard datasets. The first step assigns confidence scores to detected interactions; scores can be used to filter interactions or can be included as input to clustering algorithms. Any of the scoring approaches described earlier may be used (see section “Computational methods to help improve data quality”). Often, complexes are determined from AP‐MS data, and scoring approaches specific to this data are used.

Most prediction methods assume that complexes correspond to highly connected regions of PPI networks and cluster the networks to identify these regions. The clustering approaches can be categorized as agglomerative or divisive, and overlapping or non‐overlapping. Agglomerative approaches (Bader & Hogue, 2003; Li *et al*, 2005; Liu *et al*, 2009; Wang *et al*, 2009; Nepusz *et al*, 2012) start with seeds—individual nodes or cliques—and expand them into larger clusters by adding single nodes or merging with other clusters. Divisive approaches (van Dongen, 2000; Pu *et al*, 2007; Friedel *et al*, 2009) start with an entire network and partition it into highly connected regions. Overlapping approaches (Wang *et al*, 2009; Nepusz *et al*, 2012) allow nodes to be members of multiple clusters, to reflect overlap between complexes, while non‐overlapping approaches (van Dongen, 2000; Bader & Hogue, 2003; Liu *et al*, 2009) assign nodes to single clusters. Some methods (Wu *et al*, 2009; Leung *et al*, 2009; Srihari *et al*, 2010; Chin *et al*, 2010) try to identify core and attachment sections of complexes.

Several methods combine network clustering with information about protein function, orthology, or structure. To increase the reliability of predicted complexes, these methods look for clusters whose members have similar functions (King *et al*, 2004; Li *et al*, 2007), highly conserved orthologs in the same set of species (i.e., the complex is conserved as a functional unit (Sharan *et al*, 2005; Hirsh & Sharan, 2007)), and protein structures enabling simultaneous interactions with multiple complex members (Ozawa *et al*, 2010; Jung *et al*, 2010). Several surveys (Brohée & van Helden, 2006; Vlasblom & Wodak, 2009; Li *et al*, 2010; Srihari & Leong, 2013) evaluated complex prediction methods by comparing their results against experimentally determined complexes. The Markov Cluster Algorithm (van Dongen, 2000; van Dongen & Abreu‐Goodger, 2012) was found to be a top clustering method in three surveys (Brohée & van Helden, 2006; Vlasblom & Wodak, 2009; Li *et al*, 2010), and integration of network clustering with other information significantly improved performance (Srihari & Leong, 2013).

### Identifying interaction conditions

Understanding how PPIs produce specific phenotypes requires information on their context: when, where, and under what conditions interactions occur. Computational methods can help determine this information by text mining of the PubMed database (Chowdhary *et al*, 2012), or more commonly, by integrating transcriptomic and other data with PPI networks. Usually, these methods aim to identify cell types, tissues, and disease states in which interactions occur.

Direct evidence for interactions occurring in a given cell type or tissue is often unavailable since PPI detection is typically done in yeast cells or common cell lines. By contrast, HT gene expression data are available for a wide variety of organisms, cell types, tissues, and conditions (Barrett *et al*, 2013; Kolesnikov *et al*, 2015). A common approach for assigning PPIs to tissues is to check whether the genes encoding an interacting protein pair are both expressed in a tissue (Bossi & Lehner, 2009; Lopes *et al*, 2011). Proteomics data (Uhlen *et al*, 2015) are less extensive but can be used analogously. The TissueNet (Barshir *et al*, 2013) database uses both gene and protein expression data to assign PPIs to tissues. Assigning tissues on the basis of gene or protein expression has limitations; absence of gene expression may not indicate absence of protein expression, and presence of gene or protein expression may not mean that proteins interact. Also, since this approach estimates the presence or absence of proteins, it can only indicate whether all interactions involving a protein are absent, but not whether a specific interaction is absent. Correlation of gene expression profiles can provide information on specific interactions; a pair of genes with correlated expression profiles in a tissue or cell type may have interacting protein products (Camargo & Azuaje, 2007).

Interactions that change in disease or other conditions can be identified by similar approaches. An interaction may be disease‐related if the two encoding genes are both expressed only in the disease state (or are upregulated in the disease state; Ideker *et al*, 2002), or the genes have correlated expression profiles in disease states (Camargo & Azuaje, 2007; Guo *et al*, 2007; Xiao *et al*, 2012). Differential correlation of gene expression profiles can provide more specific information about an interaction: if two genes have significantly different correlation levels in two conditions, then the interaction of their protein products may change between conditions (Lin *et al*, 2010; Yoon *et al*, 2011; Zhang *et al*, 2012b; Yu *et al*, 2013).

### Integrating PPI networks with other omics data

Integrating PPI networks with other omics data, such as genomic, transcriptomic, and proteomic, is essential for understanding the molecular basis of phenotypes. Just as gene expression data can provide context for PPIs, and link them with specific conditions, PPI networks can provide context for other data, and link it with phenotype.

One of the most common types of integration combines PPI networks and gene‐phenotype data, to uncover relationships between genes and diseases. Goh *et al* (2007) showed that essential genes and disease genes have distinct network properties: essential genes tend to encode hub proteins, while disease genes encode proteins in the periphery of the network. Genes implicated in similar diseases tend to encode proteins that are close in PPI networks—either interacting directly (Goh *et al*, 2007; Schadt, 2009) or members of the same complex (Lage *et al*, 2007), pathway (Wood *et al*, 2007), or subnetwork (Lim *et al*, 2006). Based on this idea, it is possible to identify novel disease genes by mapping known disease‐associated genes to nodes in PPI networks, and applying random walk (Kohler *et al*, 2008; Smedley *et al*, 2014), network flow (Yeger‐Lotem *et al*, 2009; Chen *et al*, 2011), label propagation (Lee *et al*, 2011a), or other related algorithms (Vanunu *et al*, 2010; Winter *et al*, 2012). Random walk algorithms have been shown to be especially effective (Navlakha & Kingsford, 2010).

Integrating PPI networks with protein–DNA interactions, gene expression, phenotype, and drug information can provide insights into disease and drug mechanisms and can help identify new treatments. PPI networks combined with protein–DNA interactions have been used to model cellular regulatory networks—identifying regulatory circuits (Yeger‐Lotem *et al*, 2004) and signaling‐regulatory pathways (Ourfali *et al*, 2007). More recently, networks were used to predict disease mechanisms by modeling pathogen induced perturbations (Gulbahce *et al*, 2012), and effects of node or edge removal (Zhong *et al*, 2009; Sahni *et al*, 2013). Networks have also been effective for developing treatments: identifying drug targets (Yeh *et al*, 2012; Emig *et al*, 2013), understanding drug mechanism of action (Perez‐Lopez *et al*, 2015), predicting side effects (Huang *et al*, 2013b), predicting drug–drug interactions (Huang *et al*, 2013a), and characterizing drug‐regulated genes and toxicity (Kotlyar *et al*, 2012).

### Biological validations

Direct experimental validation of the biological relevance of interactions is the final important step in any interactome mapping project. While complete validation of all novel interactions detected is seldom possible within the context of a single study, demonstrating that a particular interactome provides information of practical biological importance can be done by further analysis of a representative subset of interactions.

Selection of the subset of interactions to study is highly situational, and will depend largely on the nature of the proteins being investigated and the information currently available about them, as well as the size of the interactome and the specific goals of the study. For example, if studying the interactions of a protein whose mutation is known to be associated with disease, interactions which differ between the WT and mutant forms of the protein would likely be highly informative candidates for initial validation. Interactions involving members specifically associated with a given process of interest, which have not been previously demonstrated to interact, also represent a good starting point. Integrating the interactome with other datasets, combined with various predictive algorithms (as described above), is valuable in this selection process, and can help identify candidates based on a more complex range of user‐defined criteria.

The specific validation experiments to be performed also vary on a case‐by‐case basis. Typical initial characterization experiments involve disrupting the level of individual members of an interaction pair (e.g., by gene deletion/knockdown or overexpression), and then looking for changes in the properties or function of the other member. For example, if studying the interactions of a particular receptor, one could investigate the effect of altering gene expression levels of identified interactors on downstream signaling cascades controlled by the receptor. Alternatively, effects on protein stability, protein trafficking, posttranslational modification, or responsiveness to known ligands/substrates could be probed. Investigations can be as specific as centering on molecular effects on individual proteins, or more broadly explore general phenotypic change (e.g., increased sensitivity to particular drugs, inability to grow under certain conditions). Mutational analysis of proteins can also be useful in identifying regions important for mediating and regulating interactions. For examples of proteomics screens followed by functional follow‐ups, we refer the reader to several recent studies (Babu *et al*, 2012; Snider *et al*, 2013; Petschnigg *et al*, 2014).

The importance of performing these validations cannot be understated, as they provide a clear demonstration of the usefulness of any newly generated interactome in providing a solid starting point for the identification and characterization of biologically important processes. If an interactome cannot easily provide this information, then it is unlikely to be of widespread value to the scientific community, and further improvements are necessary. It is also critical to note that carrying out proper validations may represent a significant effort, and researchers must take this into account when planning and implementing any interactome mapping project, regardless of scale.

## Concluding remarks

High‐throughput PPI mapping and analysis enables researchers to generate data and investigate biological processes on a previously unprecedented scale (Yao *et al*, 2015). Selecting and implementing the method best suited for a particular biological question can be a significant challenge, however, one which is further complicated by the emergence of an ever increasing number of new interaction proteomics technologies. Here, we have presented the general principles of the most frequently used PPI methods and have highlighted the advantages and limitations of each, as well as provided a summary of available bioinformatics approaches and resources for use in the interpretation of interactome data. It is our hope that this review serves as a useful guide to “wet” and “dry” laboratory methodologies and the analytical tools required to properly make use of these exciting approaches, and will help more scientists actively employ them in their research efforts.

## Conflict of interest

The authors declare that they have no conflict of interest.
